# Comprehensive evaluation of genetic variation in *S100A7 *suggests an association with the occurrence of allergic rhinitis

**DOI:** 10.1186/1465-9921-9-29

**Published:** 2008-03-28

**Authors:** Malin Bryborn, Christer Halldén, Torbjörn Säll, Mikael Adner, Lars Olaf Cardell

**Affiliations:** 1 Laboratory of Clinical and Experimental Allergy Research, Department of Otorhinolaryngology, Malmö University Hospital, Lund University, Malmö, Sweden; 2 Department of Clinical Chemistry, Malmö University Hospital, Malmö, Sweden; 3 Department of Cell and Organism Biology, Lund University, Lund, Sweden; 4 The National Institute of Environmental Medicine, Karolinska Institutet, Solna, Sweden; 5 Department of Otorhinolaryngology, Karolinska Institutet, Huddinge, Sweden

## Abstract

**Background:**

S100A7 is a calcium-binding protein with chemotactic and antimicrobial properties. S100A7 protein levels are decreased in nasal lavage fluid from individuals with ongoing allergic rhinitis, suggesting a role for S100A7 in allergic airway inflammation. The aims of this study were to describe genetic variation in *S100A7 *and search for associations between this variation and allergic rhinitis.

**Methods:**

Peripheral blood was collected from 184 atopic patients with a history of pollen-induced allergic rhinitis and 378 non-atopic individuals, all of Swedish origin. DNA was extracted and the *S100A7 *gene was resequenced in a subset of 47 randomly selected atopic individuals. Nine polymorphisms were genotyped in 184 atopic and 378 non-atopic individuals and subsequently investigated for associations with allergic rhinitis as well as skin prick test results. Haplotypes were estimated and compared in the two groups.

**Results:**

Thirteen polymorphisms were identified in *S100A7*, of which 7 were previously undescribed. rs3014837 (G/C), which gives rise to an Asp → Glu amino acid shift, had significantly increased minor allele frequency in atopic individuals. The major haplotype, containing the major allele at all sites, was more common in non-atopic individuals, while the haplotype containing the minor allele at rs3014837 was equally more common among the atopic individuals. Additionally, heterozygotes at this site had significantly higher scores in skin prick tests for 9 out of 11 tested allergens, compared to homozygotes.

**Conclusion:**

This is the first study describing genetic variation, associated with allergy, in *S100A7*. The results indicate that rs3014837 is linked to allergic rhinitis in our Swedish population and render S100A7 a strong candidate for further investigations regarding its role in allergic inflammation.

## Introduction

The upper airways are relatively easy to access and offer the opportunity for allergen provocation, in conjunction with repeated sampling and measurements, with a minimum of discomfort and risk for the patient [1,2]. This has prompted us to use allergic rhinitis as an experimental model when searching for suitable mediators to target in allergic airway inflammation. Using 2-dimensional gel electrophoresis in combination with mass spectrometry we have been able to identify 6 novel proteins in nasal lavage fluid [3]. One of these proteins, S100A7, also called psoriasin, appeared to be of special interest since it was found to be markedly down-regulated in patients with symptomatic allergic rhinitis [3].

S100A7 belongs to the large family of S100 proteins, which all have calcium-binding properties. The functions of secreted S100A7 are poorly investigated. The idea that S100A7 might have a role in allergic rhinitis is supported by its potent chemotactic effects on T lymphocytes and neutrophils [4]. Originally, S100A7 was identified in keratinocytes from psoriatic patients, where it was found to be highly up-regulated [5]. Thus, S100A7 was initially thought to be a specific marker for psoriasis, thereby the alternative name psoriasin, but it was soon found to play a role also in atopic eczema [6,7]. The latter further corroborating its newly identified involvement in allergic airway inflammation.

To further establish S100A7 as a factor in allergic airway inflammation, the present study was designed to describe the level and pattern of genetic variation in the *S100A7 *gene and to search for associations between this variation and allergic rhinitis.

## Methods

### Subjects

Blood samples from 184 patients (80 female, 104 male) with symptomatic birch and/or grass pollen induced allergic rhinitis and 378 healthy individuals (163 female, 214 male), serving as controls. The median (range) age of patients and controls was 32.5 (18–62) and 46 (19–69). The diagnosis of birch and/or grass pollen induced allergic rhinitis was based on a positive history of intermittent allergic rhinitis for at least 2 years and a positive skin prick test (SPT) or Phadiatop test (Pharmacia Upjohn, Uppsala, Sweden) to birch and/or grass.

All patients were classified according to the ARIA criteria [8], as having severe symptoms (itchy nose and eyes, sneezing, nasal secretion and nasal blockage) during pollen season and they had all been treated with antihistamines and nasal steroids during pollen seasons previous years. Controls had no history of allergic rhinitis or any other atopic disease. Both patients and controls were of Caucasian origin, with both parents born in Sweden. The study was approved by the Ethics Committee of the Medical Faculty, Lund University, and written informed consent was obtained from all subjects.

### Skin prick test

Skin prick tests (SPT) were performed with a standard panel of 11 common airborne allergens (ALK, Copenhagen, Denmark) including pollen (birch, timothy, mugwort and ragweed), house dust mites (*D. pteronyssimus *and *D. farinae*), molds (*Cladosporium *and *Alternaria*) and animal allergens (cat, dog and horse). SPT were performed on the volar side of the forearm with saline buffer as negative and histamine chloride (10 mg/ml) as positive control. All patients presented a wheal reaction diameter >3 mm towards birch or timothy (grass) in SPT (roughly corresponding to a 3+ or 4+ reaction when compared with histamine [9]) or a positive Phadiatop test, with at least class 2 in subsequent test with specific allergens. Approximately 44% of the patients were positive for birch and/or grass only, while ~31% were positive (≥ 2) for 1–2 additional allergens, ~20% for 3–4 and ~4% for ≥ 5 additional allergens. Controls had a negative SPT or Phadiatop test. The score used for association analysis is defined as the size of the wheal reaction in relation to histamine, i.e. 0–6.

### DNA sequencing

Genomic DNA was extracted from whole blood using QIAamp DNA Blood Mini Kit (Qiagen, Hilden, Germany). A subset of 47 randomly selected individuals with allergic rhinitis (23 female and 24 male, with median (range) age of 35 (20–61)) were used for sequencing of the putative promoter region, all coding regions and flanking intronic sequences. All primer DNA sequences are listed in Additional file 1. Samples were sequenced using Big Dye Terminator chemistry, ver. 3.1 on an ABI 3730 sequencer (Applied Biosystems, Foster City, USA). The sequence data was assembled and compared using SeqScape v2.5 (Applied Biosystems). All automatically identified candidate heterozygotes were confirmed manually and most polymorphisms were subsequently confirmed by independent genotyping.

### Genotyping

SNP genotypes were determined using the Sequenom MassARRAY MALDI-TOF system. The system analyzes allele-specific primer extension products using mass-spectrometry. Assay design was made using the MassARRAY Assay Design ver. 2.0 software (Sequenom Inc, USA). Primers (Additional file 1) were obtained from Metabion GmbH, Germany. The genotype data for rs3014837 was also analyzed using a Taqman assay (Custom TaqMan^® ^SNP Genotyping Assay ID C_736084_10, Applied Biosystems), on an ABI 7900 HT system. An 8-nucleotide indel was amplified using GeneAmp 9700 machines (Applied Biosystems) and the PCR products were resolved using capillary electrophoresis run on an ABI PRISM™ 3730 sequencer employing GeneMapper software (Applied Biosystems).

### Genetic analysis

All polymorphisms detected by DNA sequencing were assayed on a set of 2- and 3-generation families to check data quality and confirm Mendelian segregation. In order to quantify the level of genetic variation in the sequence data we calculated the expected level of heterozygosity for variable sites, *h *= 1 - *p*^2 ^- *q*^2^. Where *p *is the allele frequency of one of the alleles and *q *= 1-*p*. We also calculate *Π *which is the average number of pairwise differences among sequences and *π *which is this value per bp in the data set. In addition, *π *is also the average heterozygosity (*h*) per bp. Finally, we calculated *K/a *where *K *is the number of variable sites and *a *= Σ1/*i*, *i *= 1..*n*-1 where *n *is the number of investigated sequences. The rationale behind calculating *K/a *is the fact that if all variation in a sequence is completely neutral, then *Π *= *K*/*a *is expected. If there is directional or purifying selection *Π *<*K*/*a *is expected, whereas if balancing selection is operating for two or more alleles *Π *> *K*/*a *is expected.

A set of SNPs that produced good quality data were subsequently used to analyze 184 patients and 378 controls for associations between genetic variation in the *S100A7 *gene and allergic rhinitis. First, the genotype frequencies were calculated and tested for Hardy-Weinberg equilibrium (HWE). Next, alleles were investigated for associations with allergic rhinitis using a χ^2^-homogeneity test. Using the SNP data we also investigated the level and pattern of linkage disequilibrium and haplotype frequencies. For any two loci (sites) A and B with alleles A1/A2 and B1/B2, respectively, linkage disequilibrium was quantified through R^2 ^= D^2^/(p_A1_p_A2_p_B1_p_B2_), where p_A1 _is the allele frequency of allele 1 at locus A etc and D = p_A1B1 _- p_A1_p_B1_, where p_A1B1 _is the gamete (haplotype) frequency of A1B1. Haplotypes were estimated separately for patients and controls using the program PHASE [10].

## Results

### Discovery of polymorphisms in *S100A7*

Sequencing of the *S100A7 *gene in 47 atopic individuals resulted in identification of 13 polymorphisms, one 8-nucleotide indel and 12 SNPs (table 1). Six of the polymorphisms have been previously described (rs3124216, rs3006433, rs3014839, rs12132927, rs3014837 and rs3014836), while the remaining 7 were previously undescribed (A7:1 to A7:7). Four of the polymorphisms had minor allele frequencies (MAFs) below 5%. The two coding polymorphisms, A7:5 (first codon position) and rs3014837 (third codon position), are both non-synonymous and give rise to Lys → Gln and Asp → Glu amino acid shifts, respectively.

**Table 1:** Polymorphisms within the *S100A7 *locus

**SNP name**	**Alleles**	**Contig position**	**Location**	**Amino acid shift**	**Minor allele frequency**	**Heterozygosity**
A7:1	G/T	3924455	Promoter		0.08	0.14
A7:2	Indel	3924262	Promoter		0.21	0.33
rs3124216	T/A	3924209	Promoter		0.03	0.07
rs3006433	G/A	3924047	Promoter		0.15	0.25
A7:3	C/T	3923423	Intron 1		0.14	0.24
A7:4	A/T	3923336	Intron 1		0.14	0.24
rs3014839	G/A	3923095	Intron 1		0.19	0.31
rs12132927	T/C	3921916	Intron 1		0.05	0.10
A7:5	A/C	3921790	Exon 2	Lys → Gln	0.02	0.04
rs3014837	G/C	3921761	Exon 2	Asp → Glu	0.09	0.16
rs3014836	G/A	3921555	Intron 2		0.21	0.33
A7:6	C/T	3921498	Intron 2		0.01	0.02
A7:7	C/A	3921495	Intron 2		0.02	0.04

### Pattern of genetic variation in sequence data

As pointed out above, 2965 bp were sequenced in 47 individuals (representing 94 chromosomes) and 12 SNPs and one indel were found. The three exons cover 439 bp and included two of the SNPs. The expected heterozygosity (*h*) for the variable sites is shown in table 1. Considering the 12 SNPs only, the sum of the *h *values is 1.94, which also corresponds to *Π *for the 94 chromosomes. Per bp this will be *π *= 1.94/2965 = 0.65 * 10^-3^, which is also the expected heterozygosity per bp. Moreover, *K *= 12 and *a *= 5.126, resulting in *K/a *= 2.34, i.e. *K/a *is only moderately larger than *Π*. Thus, there is no indication that strong selection has acted on *S100A7*.

### Association between *S100A7 *polymorphisms and allergic rhinitis

To identify SNPs with patient-control allele differences, 8 SNPs were genotyped in 184 atopic individuals and 378 controls. The allele frequencies are shown in table 2. All investigated polymorphisms were in HWE, both in patients and controls. For rs3014837, there was a significant difference in MAF between atopic individuals and controls; 0.08 and 0.05 respectively (χ^2 ^= 5.15, p = 0.02). Hence, the minor allele of rs3014837 is almost twice as common in the atopic group. The analysis of this SNP was repeated using the Taqman platform. Concordance was found in 99.6% of the comparisons with only two out of 550 comparisons being discordant between the Taqman and Sequenom platforms. A7:2, the 8-nucleotide indel situated in the putative promoter region, was also analyzed for association with allergic rhinitis. There was no significant difference in MAF between the two groups; 0.16 and 0.14, respectively (χ^2 ^= 0.91) (table 2).

**Table 2:** Allele frequencies for selected polymorphisms in *S100A7*

**SNP name**	**Controls**			**Patients**			**Association test, χ^2^-value (p-value)**
		
	**Allele frequency %**		**HWE χ^2^value**	**Allele frequency %**		**HWE χ^2^-value**	
A7:1	G	97.5	0.25	G	95.6	1.32	2.79 (0.09)
	T	2.5		T	4.4		
A7:2	I	85.8	0.02	I	84.2	1.20	0.91 (0.34)
	D	14.2		D	15.8		
rs3006433	C	87.5	1.09	C	84.9	1.26	1.44 (0.23)
	T	12.5		T	15.1		
A7:3	C	90.1	1.70	C	89.9	0.01	0.01 (0.93)
	T	9.9		T	10.1		
rs3014839	C	87.5	1.00	C	85.1	1.20	1.28 (0.26)
	T	12.5		T	14.9		
rs12132927	T	91.6	1.20	T	90.4	0.33	0.45 (0.50)
	C	8.4		C	9.6		
A7:5	A	99.1	0.03	A	98.6	0.04	0.45 (0.50)
	C	0.9		C	1.4		
**rs3014837**	**G**	**95.2**	**1.68**	**G**	**91.8**	**0.59**	**5.15 (0.02)**
	**C**	**4.8**		**C**	**8.2**		
A7:7	C	98.8	0.06	C	99.2	0.01	0.33 (0.57)
	A	1.2		A	0.8		

I = insertion, D = deletion

SNP with significant association test result (p < 0.05) is in bold.

### Linkage disequilibrium and haplotype frequencies

The pattern of linkage disequilibrium (LD) across *S100A7 *is shown in table 3. A moderately complex pattern emerges, two SNPs, rs3006433 and rs3014839, show almost complete LD. These two SNPs show moderately high LD to A7:2 and rs3014837. A7:1 and A7:3 appear to be associated, whereas A7:5 and A7:7 individually appear to be in equilibrium with all other investigated polymorphisms. Given the overall distance of only 2960 bases between A7:1 and A7:7, the level of LD is expected to be rather high for this region. This is clearly not found in our data.

**Table 3:** LD pattern across the *S100A7 *gene

	A7:1	A7:2	rs3006433	A7:3	rs3014839	rs12132927	A7:5	rs3014837	A7:7
A7:1									
A7:2	0.006								
rs3006433	0.005	**0.574**							
A7:3	**0.290**	0.016	0.017						
rs3014839	0.005	**0.574**	**0.992**	0.017					
rs12132927	0.003	0.012	0.015	0.008	0.012				
A7:5	0.000	0.005	0.007	0.001	0.007	0.001			
rs3014837	0.002	**0.224**	**0.377**	0.007	**0.378**	0.006	0.011		
A7:7	0.000	0.002	0.002	0.000	0.002	0.001	0.004	0.001	

Moderate to high LD-values (R^2^) are in bold.

Haplotype frequencies were estimated separately in patients and controls (table 4). Two haplotypes stand out as differing in frequency between the two groups. One is the haplotype which carries the major allele in all sites ('1' in table 4), which is more common in controls. This is also by far the most common single haplotype. The other is haplotype no '5' in table 4 which is equally more common among the patients. This haplotype has two interesting features: 1) The minor allele at rs3014837 is almost completely associated to this haplotype, i.e. the allele frequency difference at rs3014837 observed above is at the same time a haplotype frequency difference. 2) Haplotype no. '5' carries four minor alleles which is the highest number of minor alleles of any haplotype appearing in the analysis.

**Table 4:** Estimated haplotype frequencies in patients and controls

SNP	A7:1	A7:2^†^	rs3006433	A7:3	rs3014839	rs12132927	rs3014837	A7:7	Frequency (%)		
										
Haplotype*									Controls	Patients	Difference
1	G	I	C	C	C	T	G	C	63.5	60.1	3.4
2	G	I	C	C	C	**C**	G	C	8.1	9.5	-1.4
3	G	I	C	**T**	C	T	G	C	7.2	5.7	1.5
4	G	**D**	**T**	C	**T**	T	G	C	6.6	5.5	1.1
5	G	**D**	**T**	C	**T**	T	**C**	C	3.6	7.1	-3.5
6	G	**D**	C	C	C	T	G	C	3.4	3.0	0.4
7	**T**	I	C	**T**	C	T	G	C	2.5	4.4	-1.9
8	G	I	C	C	C	T	G	**A**	1.2	0.8	0.4
9	G	I	**T**	C	**T**	T	G	C	1.2	1.6	-0.4
10	G	I	**T**	C	**T**	T	**C**	C	1.0	0.6	0.4

* Only haplotypes estimated to be present in at least one individual are listed. This corresponds to 98.3% of all haplotypes present in both patients and controls. A7:5 is not included since it was estimated by PHASE to appear only on haplotypes with lower frequencies. Minor alleles are in bold.

^† ^I = insertion, D = deletion

### Association between genotype in patients and SPT results

The Kruskall-Wallis non-parametric test was used to test for effect of genotype on the level of allergy, as scored in skin prick test, among the patients. All combinations of polymorphisms and allergens were tested, which means that a total of 99 tests were performed of which six tests yielded a p-value below 0.05. Under an overall null-hypothesis of no effects, this is the approximate number of tests expected to show a p-value below 0.05. However, four of the p-values are less than one percent. Using a Poisson approximation, the probability to obtain four or more p-values < 0.01 is less than two percent. Thus, our conclusion is that the overall null-hypothesis of no effects of any polymorphism can be rejected. The lowest p-value is obtained for the combination rs3014837 * "Alternaria", which is interesting since rs3014837 was also found to have the largest difference in allele frequency between patients and controls.

The genotype distribution at rs3014837 among patients that were given a SPT score for "Alternaria" was 91 GG, 18 GC and 2 CC, i.e. a highly skewed distribution. Thus, the relevant difference is that between GG and GC individuals. It is then noteworthy that the heterozygotes, which are overrepresented among the atopic individuals, also have a higher average score for "Alternaria" among the atopic individuals. When the genotypic means of rs3014837 for all allergens are compared, it is found that GC individuals have a higher mean than GG individuals in 9 out of 11 cases. This corresponds to a p-value of 0.033 in a one-sided sign-test, and further strengthens the hypothesis that genetic variation in *S100A7 *is related to allergic rhinitis.

## Discussion

The present study has revealed a SNP (rs3014837) that is associated with the occurrence of allergic rhinitis. When comparing 184 atopic with 378 control individuals a significant allele frequency difference was detected (0.08 versus 0.05, χ^2 ^= 5.15, p = 0.02). The minor allele is more common in the atopic group and is to a major part present on one specific haplotype, i.e. the allele frequency difference is at the same time a haplotype frequency difference. The association is corroborated by the fact that the SPT scores are significantly higher for heterozygotes compared to homozygotes at this locus for 9 out of 11 allergens tested. It should be emphasized that although there was a 10 year age difference between patients and healthy controls this does not seem to affect the outcome in the association test. We have done this test when the material was matched according to age and gender as well, with similar results (data not shown). Due to the power reduction appearing when using the matched material we have chosen to use the original population.

It is well known that the development of allergic disease is a complex process, influenced by interactions between numerous environmental and genetic factors [11]. Although genetic predisposition clearly is involved, the nature of this predisposition is still debated. No single gene has been found responsible for the development of allergic disease, thus interaction between several different genes, each with little to modest effect, is more likely. A number of genes with association to allergic disease have been reported [12,13]. The majority of studies are however for asthma phenotypes. In 2006, Ober and Hoffjan presented a list of 118 genes that had been associated with asthma or atopy-related traits. However, only 23 genes had been investigated for association with the phenotype allergic rhinitis [13].

The *S100A7 *gene is located on chromosome 1q21 [14], within a cluster of genes belonging to the *S100 *gene family [15]. This gene family consists of approximately 24 genes, of which 18 are situated on chromosome 1q21 [16]. The genomic organization of *S100A7 *was characterized by Semprini et al. in 1999 [17]. The gene is 2.7 kb large and consists of three exons and two introns. The first exon is untranslated, while exons two and three are coding for the N- and C-terminal EF-hands, respectively. In addition, a 744-bp promoter sequence is located in the 5'-UTR region [17].

A total of 13 polymorphisms were identified in *S100A7*, 7 of which have previously not been described. The gene was resequenced in 47 individuals, which means that the detection rate for SNPs with a minor allele frequency of ≥ 5% is approximately 0.99, and the corresponding number for SNPs with a minor allele frequency of 1% is 0.87 [18]. Hence, we have described a major part of the genetic variation for *S100A7 *in our population. The level of LD that is observed in S100A7 is clearly lower than what is generally observed in the HapMap data within such a limited part of the genome. The LD pattern of a region is influenced by a number of factors, one important determinant being the amount of recombination per physical unit. One possibility is thus that *S100A7 *is situated in a region with a high level of recombination. Comparing with HapMap data we see that *S100A7 *is located in a gene-rich region with recombinational hotspots fairly close on both sides. The observed level of LD is fairly low confirming our results in this respect. In addition, the rs3014837 SNP is detected exclusively in the European and not in the Yoruban, Chinese or Japanese population samples of the HapMap project. Comparing this position in the human genome with the corresponding position in the genome of our closest relative, the chimpanzee (*Pan troglodytes*), we found that the common allele was G in both species. The same was true for the rhesus monkey (*Macaca mulatta*). Thus, the G allele is most likely the ancient allele being present in our monkey relatives and the disease-associated C allele may have arisen in the European population.

The rs3014837 SNP gives rise to an Asp → Glu shift at amino acid position 28. This SNP is located in exon 2 and is coding for the N-terminal EF-hand. In contrast to most of the other S100 proteins, S100A7 is not able to bind calcium in this EF-hand [19]. The structure of S100A7 contains five α helices which have been named I-IV + II'. The N-terminal EF-hand is made up by helix I and II [19]. Amino acid 28 is positioned right before the first amino acid of helix II and this amino acid is a conserved lysine that has been reported to be critical for calcium-binding [20]. Amino acid shifts in this region might give rise to functional changes that can affect the ability to bind calcium. However, since rs3014837 gives rise to a shift between aspartic and glutamic acid, which both are acidic amino acids, this can not be considered a dramatic change, and consequently it is difficult to predict the functional effects of this SNP. Functional studies are necessary to answer this question.

Although allergic rhinitis primarily affects the upper airways, it also has systemic manifestations, and it is well known that allergic rhinitis is closely related to asthma [21,22]. Thus, these two atopic phenotypes probably share some of their genetic background. However, only 17 of the genes listed in [13] were found to be associated with the rhinitis phenotype. Very few of these studies have been replicated in other populations and may therefore to some extent be spurious findings. The lack of replication is true also in our study. Nevertheless, the altered levels of S100A7 detected in patients with allergic rhinitis and atopic eczema, respectively, suggest that S100A7 is involved in allergic inflammation and in the current study we have found a SNP that gives rise to an Asp → Glu amino acid shift that is associated with allergic rhinitis.

## Conclusion

The S100A7 protein has previously been suggested to play a role both in innate immunity and in allergic inflammation [6,7,23] and we have detected marked differences in the levels of this protein in nasal lavage fluid from patients compared to controls [3]. The findings in the present study indicate that certain genetic variation in this gene is influencing the occurrence of allergic rhinitis. Altogether, this renders the S100A7 protein a good candidate for further studies in relation to allergic inflammation.

## Competing interests

The author(s) declare that they have no competing interests.

## Authors' contributions

MB and CH coordinated the study. TS performed the majority of genetic analyses. All authors participated in the design, interpretation of data, drafting of the manuscript and they have all approved the final text.

## Supplementary Material

Additional file 1 Sequencing and genotyping primers for *S100A7*. Contains primer sequences for sequencing and genotyping of *S100A7*. Click here for file
