# Supplementary material for: Comprehensive evaluation of genetic variation in S100A7 suggests an association with the occurrence of allergic rhinitis
Source: Respir Res. 2008 Mar 28;9(1):29. doi: 10.1186/1465-9921-9-29 (PMC2335106; doi:10.1186/1465-9921-9-29)
Supplement: Additional file 1 — Sequencing and genotyping primers for S100A7. Contains primer sequences for sequencing and genotyping of S100A7. [file 1465-9921-9-29-S1.doc]

### Additional file 1. Sequencing and genotyping primers for *S100A7*

### Sequencing primers

| ***S100A7*** | **Position**  **(5´-3´)^*^** | **Forward^†^** | **Reverse^‡^** |
| --- | --- | --- | --- |
| **1 (5´UTR)** | 3924753-3924093 | GCCATCACATGGGGAACT | TGTGCTATAGGTCTTGGGTCA |
| **2 (Promoter)** | 3924131-3923391 | ATAGGGCAGACCCTGATGTG | CAGGCACTGGGCACTTCTA |
| **3 (Exon 1)** | 3923596-3923001 | CACCTGATGTGGGGCAAG | GCCAGGGCTATGCTGTGTC |
| **4 (Exon 2)** | 3921937-3921285 | CCCTCCTTCCAGCACTCC | TTTCAGATTTGCTAATGAGTCTTG |
| **5 (Exon3)** | 3921106-3920355 | GCTGGGACGGGAAAGAGT | TGGTGGCTGCATTTAGGG |

^*^ Positions were according to GenBank build 35, contig no. NT_004487.17

^†^ Forward primers were tailed with TGTAAAACGACGGCCAGT

**^‡^** Reverse primers were tailed with CAGGAAACAGCTATGACC

**Genotyping primers**

| **SNP name** | **Forward^*^** | **Reverse^*^** | **Massextend primer** |
| --- | --- | --- | --- |
| A7:1 | GATTCCCATGCCTTCCCAAC | GTGCCCTTGGCTATAATGTG | CATGCCTTCCCAACCCTCTT |
| A7:2^†^ | CCTACCTGGGTCAGCCTTTC | CCTCACAGAAGCCCTGGAAT | - |
| rs3006433 | TTGATTCAGGCTTTTCTGAG | GGAGAAGTTGAGATGTTCCC | TCAGGCTTTTCTGAGTCCATAT |
| A7:3 | CTGGGCACTTCTAGAAAACG | CTCATCCTTCTACTCGTGAC | CTTCTAGAAAACGCAAAGA |
| rs3014839 | TCCCGGTTGCTAAATCCCCT | AGAGCCTCCAAAGCATTCAC | ATCCCCTCCCCAGCTCC |
| rs12132927 | TCTGTCCTCAGCCCTCCTTC | GCTTTCAAAAAGCCTTCAGG | CCTTCCAGCACTCCCAA |
| A7:5 | AATCTTGTCATCACGTCTGG | AACACTCAAGCTGAGAGGTC | CATCACGTCTGGTGTATT |
| rs3014837 | CACAGGCACTAAGGAAGTTG | AATACACCAGACGTGATGAC | GTCAGCAGGCTTGGCTT |
| A7:7 | AGAGAGAGGGTCAGTGAGTG | GTTATACAGATATAAAGGAGG | CTCAAGCTGCACACTTT |

^*^ Primers (not A7:2) were tailed with ACGTTGGATG

^†^ Forward primer was marked with fluorophore and the reverse primer was tailed with GTTTCTT
